# The reproducibility of assessment of white spot lesions adjacent to orthodontic brackets, with a quantitative light induced fluorescence digital camera at different rotations of teeth – an in vitro study

**DOI:** 10.1186/s12903-018-0667-3

**Published:** 2018-12-11

**Authors:** Nicoline C. W. van der Kaaij, Maria J. Faaij, Jacob M. ten Cate, Monique H. van der Veen

**Affiliations:** 1 Department of orthodontics, Academic Centre for Dentistry Amsterdam (ACTA), University of Amsterdam and Free University Amsterdam, Gustav Mahlerlaan 3004, 1081 LA Amsterdam, The Netherlands; 2 Department of preventive dentistry, Academic Centre for Dentistry Amsterdam (ACTA), University of Amsterdam and Free University Amsterdam, Gustav Mahlerlaan 3004, 1081 LA Amsterdam, The Netherlands

**Keywords:** Quantitative light-induced fluorescence, Fixed orthodontic appliances, Tooth demineralization, Image analysis

## Abstract

**Background:**

A quantitative light-induced fluorescence digital (QLF-D) camera is able to assess demineralizations adjacent to orthodontic brackets. Rotations of teeth during and the presence of the orthodontic appliances may influence the longitudinal follow-up of such lesions over time.

**Methods:**

Brackets were bonded on extracted teeth: 54 incisors and 31 canines. Demineralizations were formed in vitro directly cervical of the bracket. Images were captured using a QLF-D camera mounted on an optical bench, equipped with a goniometer on a turntable. The teeth were placed in the goniometer simulating buccolingual rotation (0°, 10°, 20°), the turn-table was used for mesiodistal rotations (0°, 10°, 20°). Standardized QLF-D images were made before (with and without a wire) and after debonding at combinations of aforementioned angles of rotation. The image after debonding at 0° buccolingual and 0° mesiodistal rotation served as a control.

**Results:**

The presence of a bracket resulted in a significantly higher fluorescence loss, yet a smaller lesion area (*p* < 0.05) in comparison to the control. A significant higher fluorescence loss was seen for rotations towards lingual relative to the 0° buccolingual and 0° mesiodistal rotation, while the effect was less explicit towards buccal.

**Conclusions:**

Fluorescence loss and lesion size are influenced by the angle of rotation under which the demineralization is photographed. The full extent of demineralizations is only apparent after debonding when photographed at rotations of 0° mesiodistal and up to 20° buccal. Precaution must be taken into account assessing demineralizations of patients undergoing treatment with fixed appliances when using a QLF-D camera.

**Electronic supplementary material:**

The online version of this article (10.1186/s12903-018-0667-3) contains supplementary material, which is available to authorized users.

## Background

During orthodontic treatment with fixed appliances, white spot lesions (WSL) are frequently formed around brackets. This serious problem is the result of poor oral hygiene and occurs in 23% to 97% of patients [1–5]. Early detection of such lesions is crucial to prevent progression and further decay over time.

Quantitative light-induced fluorescence (QLF) is a method widely used to detect and quantitate WSL. In this procedure, images of teeth are made with a high intensity blue-violet light, showing healthy tooth with green fluorescence. In case of a demineralization, such as a WSL, the natural fluorescence is reduced [6, 7]. The light entering such a demineralized area is highly scattered and has a lower chance of being absorbed and reemitted as fluorescence.

One of the main advantages of QLF is that lesions may be detected earlier than through conventional visual inspection [8]. Studies also demonstrate that by sharing visual QLF images with patients, and pointing out lesions, patients are motivated to improve oral hygiene [9]. Another suggested advantage of QLF for the practitioner or researcher is the ability to monitor lesions over time in patients with fixed appliances [10].

While in vivo reproducibility of QLF-assessment has been shown for non-bracketed teeth [11], the reproducibility of the assessment of WSL with a QLF digital (QLF-D) camera in patients with fixed orthodontic appliances is not known. Images captured under the same circumstances, that is using the same camera angle, can be reproducibly quantified in vitro [12–14]. However, during orthodontic treatment it is difficult to standardize capturing QLF images because of eruption and orthodontically induced movements of the teeth, specifically rotations and angulations. Moreover, the light intensity of the incident light should be the same for all parts of the lesion and surrounding tooth tissue for optimal QLF-imaging. When the lesion is adjacent to the bracket, the light path is distorted due to the bracket itself [12]. Similarly, the presence of a wire, ligature or hook attached to the bracket may interfere QLF-imaging, either by covering parts of the lesion or causing reflection of light. An accurate assessment of the lesion is further jeopardized by a lack of sufficient healthy tissue around the lesion, which is required for a correct assessment of the lesion. An in vivo study performed in non-orthodontic individuals, also revealed that lesions adjacent to the gingiva or affected by a swollen gingiva are more difficultly detected and analysed [15]. This results in a limited use of QLF for the cervical part of the teeth. Due to the above-mentioned, limiting factors, a QLF-D assessment might be less reliable for use in orthodontic patients [12].

Furthermore, camera angle is a crucial aspect in the reproducibility of WSL assessments [10, 15]. In vitro studies show that with different camera angles, WSL images can be reproduced correctly, when imaging teeth without brackets, and in turn assessed accurately for lesion area and fluorescence loss with variations in QLF camera angles of up to 20° [15, 16]. However, when the angle was increased above 20°, there were significant differences in the outcome of the QLF-parameters [15], resulting in a higher percentage of fluorescence loss and a slight reduction of the demineralization area. For teeth with brackets WSL are reproducibly assessed at white light images with lingual angles of rotation up to 20° [10].

The objective of this study was to investigate the reproducibility when using a QLF-D camera to detect and monitor the area and the fluorescence loss of WSL adjacent to orthodontic brackets. Reproducibility is assessed for different mesiodistal and buccolingual angles of rotation up to 20° of teeth with or without brackets or in case of brackets with either a hook or elastic ligature and wire. With this data, it can be evaluated whether QLF-D can be used for longitudinal monitoring of WSL in patients with fixed orthodontic appliances.

## Materials and methods

### Objective

An in vitro experiment was performed in which extracted maxillary incisors and canines with an artificial WSL cervical of an orthodontic bracket were assessed with a QLF-D camera. Fluorescence images were captured with bracket (identified as group WB), with bracket, grey elastic ligature and wire (group WE) and after debonding (group AD). The orientation of the buccal surface towards the camera was varied by rotations up to 20° along the tooth’s length axis, i.e. mesiodistal rotations, or transverse axis, i.e. tilting the tip of the crown forwards to (simulating rotations around the transverse axis towards buccal) or backwards from (lingual) the camera). These rotations were chosen as being the rotations seen in patients with a near-normal occlusion [17].

### Sample size and power calculation

To determine the sample size, a power analysis was performed based on a t-test to compare the means of two dependent groups with an alpha of 0.05 (G*Power 3.1.9.2). In comparison to incisors, the surface morphology of canines and presence of a hook on the canine-bracket are expected to have a stronger effect on caries outcomes for different angles of rotation. Thus for the canines the influence of both mesial and distal angles of rotation were assessed, resulting in 75 different angles of rotation. For the incisors only the mesial angles of rotation were assessed, resulting in 45 images per incisor. For the sample size calculation an effect size of 0.6 was assumed for canines, and an effect size of 0.45 for incisors. This resulted in a minimum sample size of 31 for the canines and 54 for the incisors.

### Procedure

Extracted teeth were collected. The selected teeth were all maxillary and had no restorations and showed no caries, discoloration or enamel defects on the surface to be studied. The teeth were polished with a cup and polishing paste (Zircate Prophy Paste, Dentsply International, York, United States). Brackets (APC II Vicory Series Low Profile metal bracket, 3 M Unitek, Monrovia, United States) were bonded (Transbond XT Light Cure Adhesive paste, 3 M Unitek, Delft, The Netherlands) without using primer or etchant. The brackets were bonded in the middle of the tooth mesiodistally and a periodontal probe was used to bond the brackets 2.5 mm from the incisal edge for the incisors and 3 mm from the incisal edge for the canines. Primer and etchant were not used to avoid demineralization effects at unwanted places. The brackets bonded to the canines had a soldered hook attached to the cervical part of the bracket. The hooks were all bonded in the same direction. The excess of composite around the bracket was removed with a microbrush and probe. Blue tape (PVC tape, Coroplast, Wuppertal, Germany) measuring two by three millimetres was attached directly cervical of the brackets on the teeth [18]. The tape covered the area where the WSL was created. The teeth were then covered with fluoride free bonding material (Clearfil SE Bond, Kuraray Dental, New York, United States), thus excluding the places where the bracket and tape were located. Next, the tape was removed and the teeth were placed in a glass container with the bracket facing up. WSL were created according to a demineralization procedure [19, 20] using methylcellulose gel and lactic acid (pH 4.6) for 17 days. This resulted in less demineralization than anticipated and thus all teeth were etched (Ultra-Etch, Ultradent, South Jordan, United States) for five minutes, on the place where the demineralization was formed in order to obtain homogeneous WSL in the range of ICDAS 1 and 2 for the assessment on QLF images. The teeth were kept in the refrigerator in distilled water, except when capturing the images. Prior to capturing the images, the teeth, but not the WSL, were polished to remove the fluoride free bonding.

### Standardized set-up and outcome measures

**Fig. 1:**
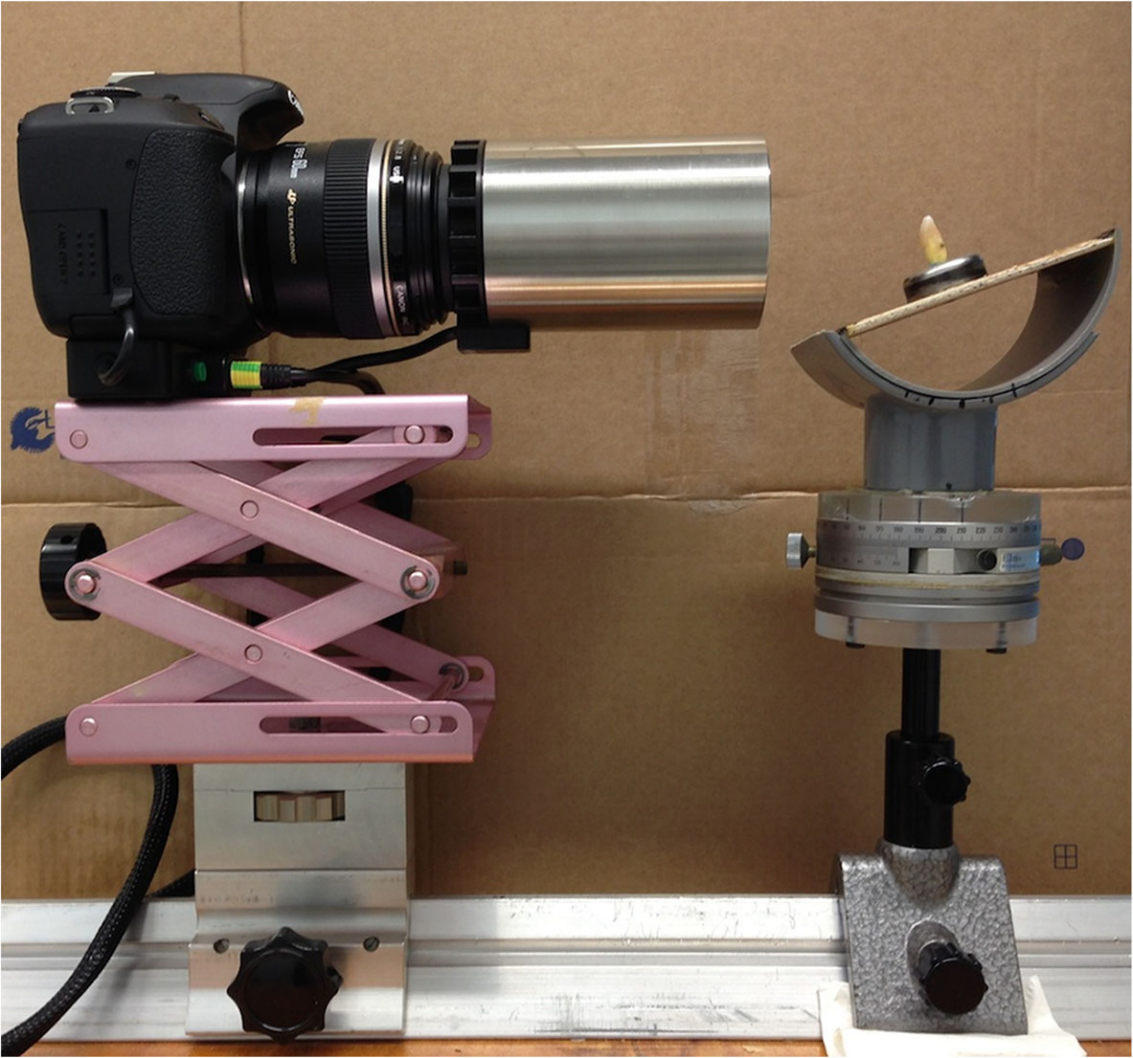
Standardized set-up. *Incisor (AD) in goniometer at 0° mesiodistal and 20° buccal*

Teeth were captured in a standardized set-up using a QLF-D camera mounted on an optical bench, which further comprised a self-constructed goniometer mounted on a turntable (Fig. 1). Teeth were placed up to seven millimetres apical of the cement-enamel junction in the goniometer, at 50 mm distance measured from the end of the tube, to simulate buccolingual (bl) tooth rotation. The turn-table mounted under the goniometer, was used to simulate mesiodistal (md) tooth rotation.

The camera focal length was fixed for the duration of the experiment. The teeth were rotated along their length axis or transverse axis (tilting the tip of the crown forwards (buccal) to or backwards (lingual) from the camera).

**Fig. 2:**
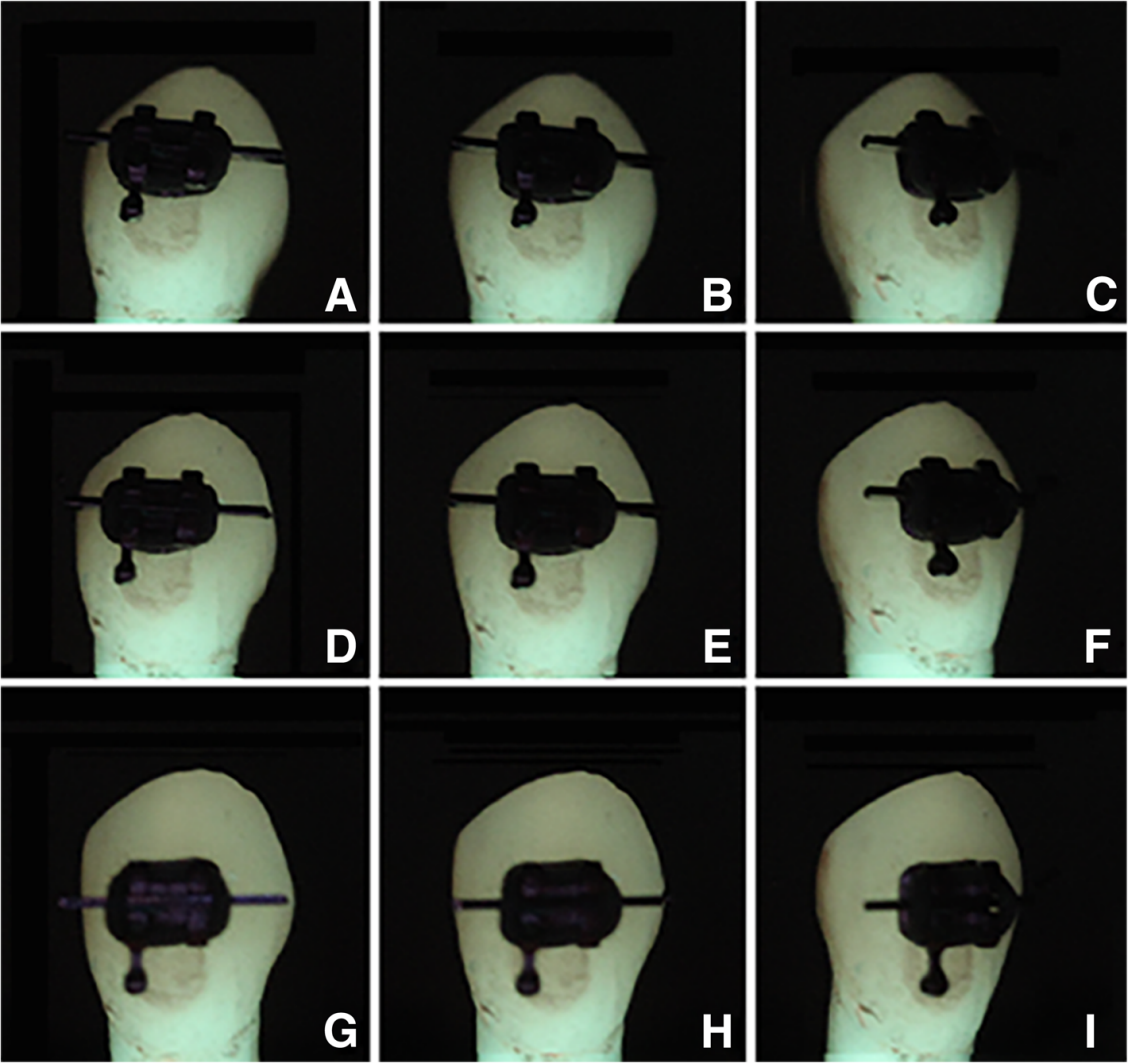
QLF photos of a canine WE at a few different angles. *A: 10°m × 10°l, B: 0°md × 10°l, C: 20°d × 10°l, D: 10°m × 0°bl, E: 0°md × 0°bl, F: 20°d × 0°bl, G: 10°m × 20°b, H: 0°md × 20°b, I: 20°d × 20°b*

The images were captured in a room with dimmed light. A box was placed over the standardized set-up to create a dark room in order to avoid bias caused by different ambient light conditions. A dark background was used to enlarge the contrast in the images and to mimic the natural situation inside the mouth. QLF-images were captured from the buccal surfaces of all teeth using the QLF-D Biluminator system (Inspektor Research Systems, Amsterdam, The Netherlands). The QLF-D camera consisted of an illumination tube (Biluminator™; Inspektor Research Systems B.V., Amsterdam) fitted on a single-lens reflex camera (Canon model 650-D, fitted with a 60 mm Macro lens; Canon Inc., Tokyo). The illumination tube is composed of a ring with 12 violet-blue LEDs (405 ± 20 nm) with filtering optics in the centre and having transmission peaks of 90% around 640 nm, 15% around 500 nm and 20% around 440 nm. Two images were made of each tooth per position with default settings; one white-light image and one QLF image. Only the QLF images were used for the assessment. The images were captured at different angles of tooth rotation: for all teeth 0°, 10° and 20° towards mesial (0°md, 10°m, 20°m); towards buccal (0°bl, 10°b, 20°b); and towards lingual (0°bl, 10°l, 20°l) and only for the canines, because of the soldered hook on one side of the bracket, also towards distal (0°md, 10°d, 20°d). Images were first captured with just brackets on the teeth (group WB), secondly with brackets and with a wire ligated with a grey elastic (NiTi 16 × 22, Dentsply Lomberg, Zoetermeer, The Netherlands) in place (group WE) (Fig. 2). Finally images were captured after debonding of the brackets (group AD) to obtain a view of the full lesion extent. This led to a total of 45 photographs per tooth for the incisors and 75 images per tooth for the canines.

### Data assessment (statistics)

Analysing WSL on the images was done using QA2 Data analysis software (version 1.26, QLF-D Research Suite, Inspektor Research Systems). The program makes a comparison between sound and demineralized enamel [21]. The comparison is based on a contour, which is drawn around the WSL. A sound patch is reconstructed through a two-dimensional linear interpolation of the sound enamel of the contour. The decrease in fluorescence is determined between the reconstructed sound and demineralized area and the mean percentage fluorescence loss (ΔF[%]) and the lesion area of the WSL are calculated.

A random selection of 10% of the teeth were analysed by two persons (MF and NK) for the inter-examiner reliability. After four weeks, examiner MF analysed the images of six incisors and three canines again for the intra-examiner reliability. For the results, the assessments of examiner MF were used in all cases. Data regarding lesion area and fluorescence loss was presented in figures for incisors and canines separate to visualize changes caused by the rotation of teeth and the direction of such changes. A t-test was used to compare images WB, WE or AD at all angles of rotation to the non- rotated control image after debonding (0°md and 0°bl AD). For the second assessment they were compared to their corresponding non-rotated control image (e.g. 0°md and 0°bl WB) (ANOVA for repeated measures; followed by t-test). For the third assessment the mesiodistal rotations combined without a buccolingual rotation were used as control image in comparison to the images with corresponding mesiodistal rotation and different buccolingual rotations (t-test). A fourth assessment compared the differences between the WB and WE images for the same angle (t-test). Both fluorescence loss ΔF[%] and area were assessed separately. All analyses were carried out using IBM SPSS Statistics 23.

## Results

The inter-examiner ICC values for ΔF[%] were 0.75 for the incisors and 0.66 for the canines. For area the ICC values were 0.76 for the incisors and 0.81 for the canines. The respective intra-examiner ICC values (examiner MF) were 0.95 for the incisors and 0.94 for the canines regarding lesion area. Regarding ΔF[%] the intra-examiner ICC values were 0.82 for the incisors and 0.81 for the canines.

Eighty-five extracted teeth were photographed. On one canine the bracket came loose during placement of the elastic ligature and wire. As a result 30 canines were used in the analysis of WE.

The presence of a bracket (both WB and WE) resulted in a significantly higher ΔF[%] and a lower lesion area (*p* < 0.05) relative to the non-rotated control image after debonding (0°md-0°bl AD). This applied to the incisors and canines, separate and combined. The canines showed overall a lower ΔF[%] and a lower lesion area relative to the incisors.

### Fluorescence loss (ΔF[%])

**Fig. 3:**
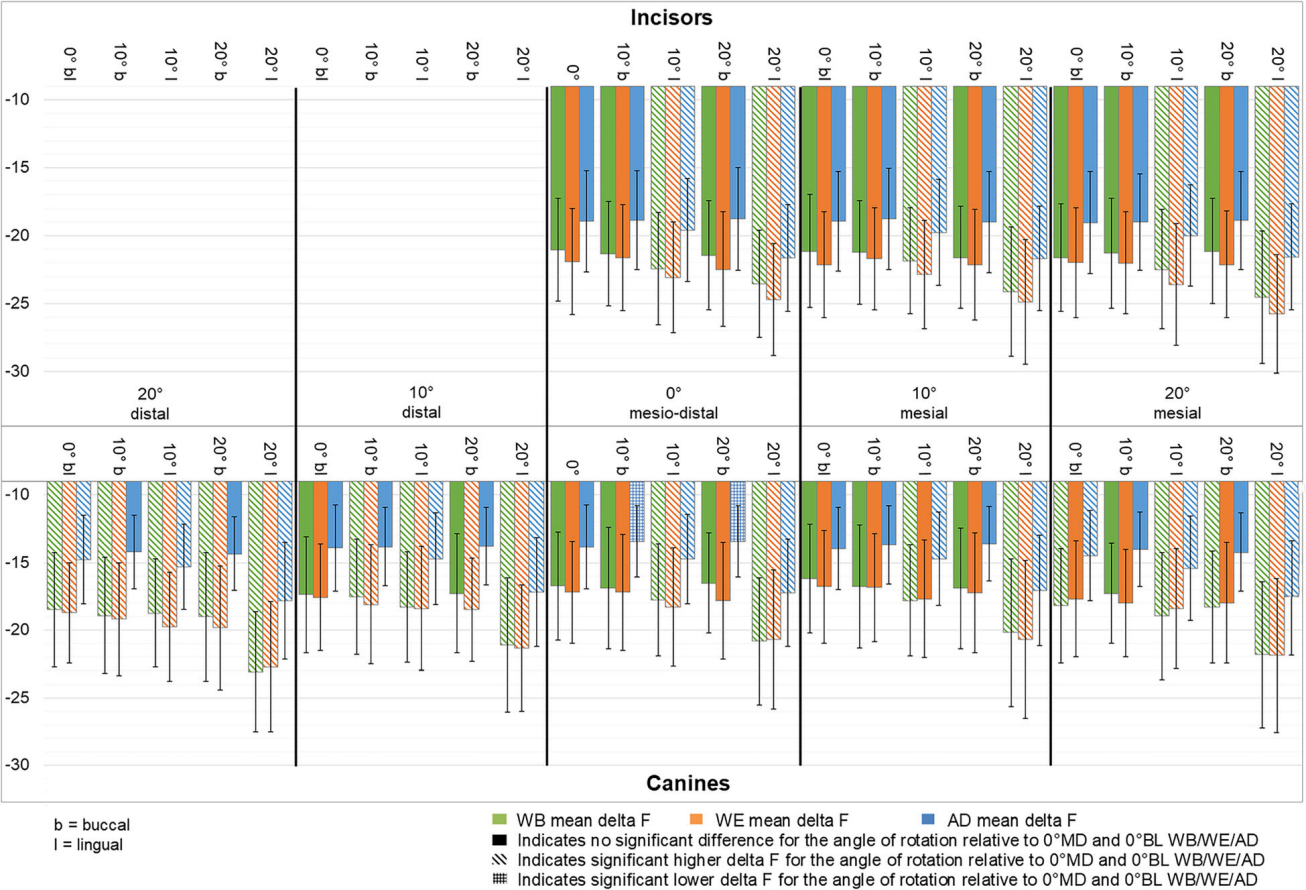
Average ΔF[%] at the different rotation angles. *Average* ΔF[%] *on the y-axis and on the x-axis the different rotation angles for the teeth photographed. The incisors are represented in the upper part and the canines in the lower part. The data are presented in green for WSL images with bracket (WB), in orange for WSL images with elastic ligature and wire (WE) and in blue WSL images after debonding (AD)*

The influence of rotation during assessment on fluorescence loss is shown in Fig. 3. In Fig. 3 striped patterns represent a significantly different fluorescence loss relative to the non-rotated control image with the corresponding colour. In Additional file 1: Table S1 and Additional file 2: Table S2 the descriptive data are presented.

#### Incisors

An increased ΔF[%] was seen for rotations towards lingual compared to the non-rotated control, showing a higher ΔF[%] with a larger angle towards lingual. This was significant for the series with bracket (WB): *F*(7.40, 391.97) = 18.75, *p* = 0.0; with elastic and ligature (WE): *F*(8.91, 472.36) = 24.50, *p =* 0.0; and without bracket (AD): *F*(6.17, 326.97) = 56.31, *p =* 0.0.

No significant differences in fluorescence loss were seen for rotations towards buccal. This significance was seen for all the lingual rotations compared to 0° mesiodistal as well as for the lingual rotations compared to the corresponding 10° or 20° mesial rotation.

#### Canines

For the canines a greater fluorescence loss was seen for the increasing angles towards lingual. Towards buccal only a larger angle towards mesial or distal showed a significance.

*Incisors and canines combined.*

Almost all angles of rotation showed a significant higher ΔF[%] between images WB and images WE when comparing the same angles of rotation.

### Area

**Fig. 4:**
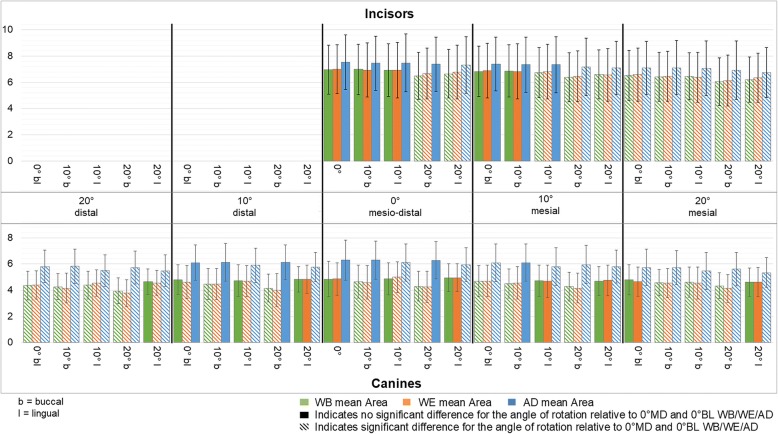
Average lesion area at the different rotation angles. *Average lesion area [mm] on the y-axis and on the x-axis the different rotation angles for the teeth photographed. The incisors are represented in the upper part and the canines in the lower part. The data are presented in green for WSL images with bracket (WB), in orange for WSL images with elastic ligature and wire (WE) and in blue WSL images after debonding (AD)*

The influence of rotations on lesion area is shown in Fig. 4. In Fig. 4 striped patterns represent a significantly different area relative to the non-rotated control image with the corresponding colour. In Additional file 3: Table S3 and Additional file 4: Table S4 the descriptive data are presented.

#### Incisors

A significantly smaller lesion area relative to that of the non-rotated control images was seen for a mesial rotation of 20° for all groups regardless of the buccolingual rotation. This trend was less pronounced at a mesial rotation of 10° and at 0° mesiodistal. After debonding all rotations of 20° lingual, irrespectively of the mesial rotation resulted in a significantly smaller lesion area relative to the control.

#### Canines

A significantly smaller lesion area relative to the non-rotated control images was seen for 50 out of 72 angles of rotation towards mesiodistal or buccolingual. When brackets were present, lesion area was influenced more for rotations towards buccal than towards lingual. After debonding the opposite was seen, all rotations towards lingual showed a significantly smaller lesion area. In case of brackets, distal rotations had more influence than mesial rotations. With brackets irrespective of the mesial rotation, rotations towards buccal resulted in smaller lesions size, while such an influence was not seen after debonding.

#### Incisors and canines combined

No significant differences in lesion areas were found between images WB and images WE when comparing the same angles of rotation.

## Discussion

The presence of orthodontic brackets with or without an elastic ligature and wire affected WSL assessment with QLF-D when compared with the situation after debonding. Even when QLF-D images of teeth with brackets were obtained under standardized conditions the ΔF[%] was overestimated, while the lesion area was underestimated. This result was not consistent with previous reports on light images, which showed that on 20 maxillary central incisors with brackets the area of WSL was reproducibly assessed for lingual rotations up to 20° in comparison to teeth without fixed appliances [10]. This discrepancy may be caused by the use of QLF images instead of light images and a bigger sample size in this study. To calculate ΔF[%] and area a comparison was made between sound and demineralized enamel [21]. Due to the presence of a bracket there was no sound enamel available for the assessment on the coronal site. Therefore this site was excluded on the contour around the WSL for the reconstructed sound patch, leading to a higher contrast between sound and demineralized enamel and, as shown in this study, resulting in a higher fluorescence loss and a smaller lesion area [15]. Besides that, the enamel in the coronal area is thicker than that of the cervical area [15].

No significant differences in lesion area, incisors and canines together, were found, for the same rotation angle of group WB relative to group WE. In contrast ΔF[%] was overestimated for almost all angles of rotation when comparing the same angle in group WE to group WB. This indicates that elastic ligatures around and wires through the bracket did not influence the measured area of the WSL, but did have an influence on ΔF[%]. An elastic ligature placed around the bracket with a wire resulted in a shadow on the WSL and hence a darker appearance of the WSL and therefore a further difference in contrast of ΔF[%] [16]. An elastic ligature around the bracket with a wire thus interfered in the analysis of WSL, due to a distorted light intensity. It is feasible that other materials used during an orthodontic treatment with fixed appliances may also create interference, such as a Kobayashi hook, a chain elastic or an overlay arch.

In this study for all groups of incisors rotations towards lingual always resulted in a significant higher ΔF[%] relative to the corresponding non-rotated control image, but rotations towards buccal did not give significant differences for ΔF[%]. This showed that ΔF[%] is overestimated for all incisors with or without brackets only towards the lingual direction. In the lingual direction thinner cervical enamel is used as a reference in the assessment of the WSL, compared to the thicker and brighter coronal enamel. When the enamel is thinner, the fluorescence is higher due to more reflection of dentin [15].

Further, this study showed that for incisors after debonding at 0°md only a lingual rotation of 20° gave a significant smaller lesion area relative to the non-rotated control image after debonding. For canines after debonding at 0°md lingual rotations relative to the non-rotated control image resulted in a significant smaller lesion area, while the angles of rotation towards buccal did not result in a significant smaller lesion area. This result is consistent with previous research which showed that the lesion area of WSL at teeth after debonding can be reproducibly measured at buccal rotations up to 20° [15, 16].

The presence of orthodontic brackets at the incisors or canines, separately or together, with or without an elastic ligature and wire affected WSL assessment. In this study all rotations resulted in a significant higher ΔF[%] and a smaller lesion area in comparison to the non-rotated control image after debonding. In the assessment of WSL the bracket with or without a soldered hook caused a higher ΔF[%] and a smaller area. This shows that a bracket with or without an attached hook makes no difference on the WSL assessment in comparison to teeth after debonding. Whilst, brackets on canines with a, at the mesial site, attached hook show more significance towards distal, regardless of the buccal or lingual angle. This means that the presence of a hook attached to the cervical part of the bracket has the largest effect on rotations towards the side opposite of the location of the hook, due to interference of the hook being projected over the WSL.

To summarize, when there is a bracket on a tooth there is less healthy tooth tissue available to use as a reference in the assessment of a WSL. Such a healthy tooth tissue reference is required to make a reliable calculation of ΔF[%] and area [12]. This resulted for all bracketed teeth in a significantly higher ΔF[%] and a smaller area for all angle combinations when compared to teeth after debonding. For teeth after debonding, the lesion area seemed to be reproducibly measured with QLF-D for rotations up to 0°md and 20°b. In such a situation there is healthy tooth tissue available on the coronal site to use as a reference in the assessment of a WSL. Furthermore, this study showed that the effect of a bracket on a tooth on the mean values for area is bigger than for ΔF[%] and that a hook attached to the bracket has the largest effect on rotations towards the side opposite of the location of the hook.

## Conclusion

A QLF-D camera can detect WSL adjacent to orthodontic brackets irrespective the presence of an elastic ligature and wire. However, ΔF[%] is overestimated and the lesion area is underestimated, when compared with teeth after debonding, at various mesiodistal and buccolingual rotations (0° and up) under which the WSL is photographed. This is due to the presence of the bracket, where a healthy tissue reference at the coronal part of the tooth is not available to determine the WSL. Furthermore, elastic ligatures and wires around or through brackets in orthodontic treatment resulted in a significant overestimation ​​of ΔF[%], but not for lesion area, both parameters compared to teeth with brackets without elastic ligature and wire. The presence of a hook attached to the cervical part of the bracket had the largest effect on these parameters under rotations towards the side opposite of the location of the hook. This implies that precaution must be taken when assessing WSL over time in patients undergoing treatment with fixed appliances using QLF-D. The images of the tooth should always be made under the same angle of rotation, with the same light intensity and for example always without a wire. The full extent of WSL developed adjacent to orthodontic brackets will only become apparent after debonding with rotations until 20° towards buccal and 0° mesiodistal. Thus the use of QLF for longitudinal follow-up of WSL is limited clinically, but QLF is very useful for demonstrating purposes, showing patients the presence of WSL, which are earlier detectable by QLF than by visual inspection [8] and showing the presence of matured plaque as red fluorescence in the images [9].

### Recommendations

QLF can be effectively used to identify and show demineralizations on teeth with or without orthodontic brackets. A suitable application is for example, the use as a preventive tool and for demonstrating purposes in the dental and orthodontic practice. In research settings for patients with fixed appliances over time QLF-D is less easily used, since too much alterations take place over time thus interfering with the measurements. Also after debonding a standardized method of monitoring lesions, such as a bite block, is recommended in research settings. Further research is needed to investigate whether the effect of rotations of teeth in patients with fixed appliances can be corrected for, for example by a percentage of over- or underestimation of ΔF[%] and area.

## Additional files

Additional file 1: **Table S1.** Descriptive data and statistical outcome for the effect of rotation on fluorescence loss (ΔF[%]) for the incisors (*n*=54). (DOCX 14 kb)

Additional file 2: **Table S2.** Descriptive data and statistical outcome for the effect of rotation on fluorescence loss (ΔF[%]) for the canines (WB: *n*=31, WE: *n*=30, AD: *n*=31 ). (DOCX 16 kb)

Additional file 3: **Table S3.** Descriptive data and statistical outcome for the effect of rotation on lesion area [mm] for the incisors (*n*=54). (DOCX 15 kb)

Additional file 4: **Table S4.** Descriptive data and statistical outcome for the effect of rotation on lesion area [mm] for the canines (WB: n=31, WE: *n*=30, AD: *n*=31 ). (DOCX 15 kb)

## Abbreviations

AD: Incisors and/or canines after debonding
b: Buccal
bl: Buccolingual
d: Distal
l: Lingual
m: Mesial
md: Mesiodistal
QLF: Quantitative light-induced fluorescence
QLF-D: Quantitative light-induced fluorescence digital
WB: Incisors and/or canines with bracket
WE: Incisors and/or canines with bracket, grey elastic ligature and wire
WSL: White spot lesions
