# Supplementary material for: The reproducibility of assessment of white spot lesions adjacent to orthodontic brackets, with a quantitative light induced fluorescence digital camera at different rotations of teeth – an in vitro study
Source: BMC Oral Health. 2018 Dec 11;18:209. doi: 10.1186/s12903-018-0667-3 (PMC6290521; doi:10.1186/s12903-018-0667-3)
Supplement: Supplementary file 1 — Table S1. Descriptive data and statistical outcome for the effect of rotation on fluorescence loss (ΔF[%]) for the incisors (n=54). (DOCX 14 kb) [file 12903_2018_667_MOESM1_ESM.docx]

| **Rotation Angle** | **WB Delta F** | | **WE Delta F** | | **AD Delta F** | |
| --- | --- | --- | --- | --- | --- | --- |
|  | **Mean (sd)** | **p-value comparison with 0°** | **Mean (sd)** | **p-value comparison with 0°** | **Mean (sd)** | **p-value comparison with 0°** |
| **0°md-20°l** | -23.55 (4.0) | 0.000* | -24.72 (4.1) | 0.000* | -21.65 (4.0) | 0.000* |
| **0°md-10°l** | -22.45 (4.1) | 0.000* | -23.09 (4.1) | 0.000* | -19.58 (3.8) | 0.000* |
| **0°** | -21.05 (3.8) | - | -21.92 (3.9) | - | -18.95 (3.7) | - |
| **0°md-10°b** | -21.32 (3.8) | 0.252 | -21.62 (3.9) | 0.389 | -18.89 (3.6) | 0.692 |
| **0°md-20°b** | -21.44 (4.0) | 0.208 | -22.48 (4.2) | 0.104 | -18.78 (3.8) | 0.288 |
| **10°m-20°l** | -24.14 (4.8) | 0.000* | -24.89 (4.6) | 0.000* | -21.69 (3.9) | 0.000* |
| **10°m-10°l** | -21.86 (3.9) | 0.017* | -22.88 (4.0) | 0.014* | -19.77 (3.9) | 0.000* |
| **10°m-0°bl** | -21.14 (4.2) | 0.776 | -22.16 (3.9) | 0.545 | -18.97 (3.7) | 0.861 |
| **10°m-10°b** | -21.24 (3.8) | 0.529 | -21.71 (3.7) | 0.553 | -18.76 (3.7) | 0.181 |
| **10°m-20°b** | -21.61 (3.7) | 0.104 | -22.17 (4.1) | 0.507 | -19.00 (3.7) | 0.747 |
| **20°m-20°l** | -24.55 (4.9) | 0.000* | -25.77 (4.4) | 0.000* | -21.56 (3.9) | 0.000* |
| **20°m-10°l** | -22.48 (4.4) | 0.000* | -23.61 (4.5) | 0.000* | -20.00 (3.7) | 0.000* |
| **20°m-0°bl** | -21.63 (4.0) | 0.129 | -22.00 (4.0) | 0.852 | -19.04 (3.7) | 0.643 |
| **20°m-10°b** | -21.30 (4.1) | 0.538 | -22.01 (3.7) | 0.788 | -19.03 (3.6) | 0.614 |
| **20°m-20°b** | -21.14 (3.9) | 0.798 | -22.14 (3.9) | 0.528 | -18.89 (3.6) | 0.787 |
| **ANOVA for Repeated measures** | *F*(7.40, 391.97)=18.75, *p*=0.0 | | *F*(8.91, 472.36)= 24.50, *p=*0.0 | | *F*(6.17, 326.97)=56.31, *p=*0.0 | |

Additional table S1: Descriptive data and statistical outcome for the effect of rotation on fluorescence loss (ΔF[%]) for the incisors (n=54).
