# Supplementary material for: The reproducibility of assessment of white spot lesions adjacent to orthodontic brackets, with a quantitative light induced fluorescence digital camera at different rotations of teeth – an in vitro study
Source: BMC Oral Health. 2018 Dec 11;18:209. doi: 10.1186/s12903-018-0667-3 (PMC6290521; doi:10.1186/s12903-018-0667-3)
Supplement: Supplementary file 2 — Table S2. Descriptive data and statistical outcome for the effect of rotation on fluorescence loss (ΔF[%]) for the canines (WB: n=31, WE: n=30, AD: n=31 ). (DOCX 16 kb) [file 12903_2018_667_MOESM2_ESM.docx]

| **Rotation Angle** | **WB Delta F** | | **WE Delta F** | | **AD Delta F** | |
| --- | --- | --- | --- | --- | --- | --- |
|  | **Mean (sd)** | **p-value comparison with 0°** | **Mean (sd)** | **p-value comparison with 0°** | **Mean (sd)** | **p-value comparison with 0°** |
| **20°d-20°l** | -23.08 (4.4) | 0.000* | -22.72 (4.8) | 0.000* | -17.83 (4.3) | 0.000* |
| **20°d-10°l** | -18.75 (4.0) | 0.000* | -19.75 (4.0) | 0.000* | -15.31 (3.2) | 0.000* |
| **20°d-0°bl** | -18.48 (4.3) | 0.000* | -18.72 (3.7) | 0.002* | -14.77 (3.3) | 0.000* |
| **20°d-10°b** | -18.91 (4.3) | 0.000* | -19.20 (4.2) | 0.000* | -14.22 (2.7) | 0.098 |
| **20°d-20°b** | -19.01 (4.8) | 0.000* | -19.83 (4.6) | 0.000* | -14.35 (2.7) | 0.058 |
| **10°d-20°l** | -21.12 (5.0) | 0.000* | -21.34 (4.7) | 0.000* | -17.20 (4.0) | 0.000* |
| **10°d-10°l** | -18.30 (4.1) | 0.001* | -18.39 (4.6) | 0.011* | -14.72 (3.4) | 0.000* |
| **10°d-0°bl** | -17.37 (4.3) | 0.098 | -17.58 (4.0) | 0.351 | -13.93 (3.2) | 0.580 |
| **10°d-10°b** | -17.53 (4.3) | 0.013* | -18.10 (4.4) | 0.020* | -13.82 (2.9) | 0.962 |
| **10°d-20°b** | -17.28 (4.4) | 0.116 | -18.48 (3.8) | 0.002* | -13.80 (2.9) | 0.873 |
| **0°md-20°l** | -20.83 (4.7) | 0.000* | -20.70 (5.2) | 0.000* | -17.23 (4.0) | 0.000* |
| **0°md-10°l** | -17.78 (4.1) | 0.003* | -18.28 (4.4) | 0.002* | -14.75 (3.3) | 0.000* |
| **0°** | -16.74 (4.0) | - | -17.19 (3.8) | - | -13.83 (3.1) | - |
| **0°md-10°b** | -16.89 (4.5) | 0.642 | -17.21 (4.3) | 0.282 | -13.42 (2.6) | 0.016* |
| **0°md-20°b** | -16.51 (3.7) | 0.514 | -17.83 (4.3) | 0.122 | -13.43 (2.6) | 0.027* |
| **10°m-20°l** | -20.19 (5.5) | 0.000* | -20.70 (5.9) | 0.000* | -17.08 (4.1) | 0.000* |
| **10°m-10°l** | -17.81 (4.1) | 0.005* | -17.68 (4.4) | 0.230 | -14.72 (3.5) | 0.000* |
| **10°m-0°bl** | -16.20 (4.0) | 0.108 | -16.80 (4.2) | 0.296 | -13.96 (3.1) | 0.354 |
| **10°m-10°b** | -16.78 (4.6) | 0.911 | -16.86 (4.0) | 0.469 | -13.69 (2.9) | 0.405 |
| **10°m-20°b** | -16.91 (4.5) | 0.676 | -17.23 (4.5) | 0.938 | -13.62 (2.8) | 0.294 |
| **20°m-20°l** | -21.82 (5.4) | 0.000* | -21.89 (5.7) | 0.000* | -17.56 (4.3) | 0.000* |
| **20°m-10°l** | -18.95 (4.7) | 0.000* | -18.43 (4.4) | 0.020* | -15.43 (3.9) | 0.000 |
| **20°m-0°bl** | -18.20 (4.2) | 0.006* | -17.68 (4.3) | 0.281 | -14.47 (3.3) | 0.014* |
| **20°m-10°b** | -17.27 (3.7) | 0.285 | -18.00 (4.0) | 0.100 | -14.03 (2.7) | 0.462 |
| **20°m-20°b** | -18.30 (4.2) | 0.009* | -17.98 (4.5) | 0.151 | -14.24 (2.9) | 0.213 |
| **ANOVA for Repeated measures** | *F*(8.86, 265.89)=22.85, *p*=0.0 | | *F*(8.65, 250.93)=17.51,  *p*=0.0 | | *F*(4.48, 134.31)=45.30, *p*=0.0 | |

Additional table S2: Descriptive data and statistical outcome for the effect of rotation on fluorescence loss (ΔF[%]) for the canines (WB: n=31, WE: n=30, AD: n=31 ).
