# Supplementary material for: The reproducibility of assessment of white spot lesions adjacent to orthodontic brackets, with a quantitative light induced fluorescence digital camera at different rotations of teeth – an in vitro study
Source: BMC Oral Health. 2018 Dec 11;18:209. doi: 10.1186/s12903-018-0667-3 (PMC6290521; doi:10.1186/s12903-018-0667-3)
Supplement: Supplementary file 3 — Table S3. Descriptive data and statistical outcome for the effect of rotation on lesion area [mm] for the incisors (n=54). (DOCX 15 kb) [file 12903_2018_667_MOESM3_ESM.docx]

| **Rotation Angle** | **WB area** | | **WE area** | | **AD area** | |
| --- | --- | --- | --- | --- | --- | --- |
|  | **Mean (SD)** | **p-value comparison with 0°** | **Mean (SD)** | **p-value comparison with 0°** | **Mean (SD)** | **p-value comparison with 0°** |
| **0°md-20°l** | 6.66 (1.8) | 0.001* | 6.78 (2.0) | 0.039* | 7.32 (2.2) | 0.020* |
| **0°md-10°l** | 6.94 (2.0) | 0.818 | 6.93 (2.1) | 0.445 | 7.48 (2.2) | 0.564 |
| **0°** | 6.96 (1.9) | - | 7.00 (1.9) | - | 7.55 (2.1) | - |
| **0°md-10°b** | 6.98 (1.9) | 0.731 | 6.93 (2.1) | 0.330 | 7.46 (2.1) | 0.238 |
| **0°md-20°b** | 6.51 (1.8) | 0.000* | 6.68 (1.9) | 0.000* | 7.38 (2.1) | 0.179 |
| **10°m-20°l** | 6.59 (1.9) | 0.000* | 6.58 (2.0) | 0.000* | 7.09 (2.0) | 0.001* |
| **10°m-10°l** | 6.74 (1.9) | 0.008* | 6.82 (2.1) | 0.037* | 7.34 (2.1) | 0.055 |
| **10°m-0°bl** | 6.83 (1.9) | 0.148 | 6.89 (2.1) | 0.130 | 7.38 (2.0) | 0.123 |
| **10°m-10°b** | 6.86 (2.0) | 0.238 | 6.83 (2.1) | 0.061 | 7.34 (2.1) | 0.088 |
| **10°m-20°b** | 6.39 (1.9) | 0.000* | 6.46 (2.0) | 0.000* | 7.18 (2.2) | 0.001* |
| **20°m-20°l** | 6.22 (1.7) | 0.000* | 6.35 (1.9) | 0.000* | 6.76 (1.9) | 0.000* |
| **20°m-10°l** | 6.45 (1.9) | 0.000* | 6.38 (1.9) | 0.000* | 7.07 (2.1) | 0.000* |
| **20°m-0°bl** | 6.53 (1.9) | 0.000* | 6.59 (2.0) | 0.000* | 7.09 (2.0) | 0.000* |
| **20°m-10°b** | 6.41 (1.9) | 0.000* | 6.48 (1.9) | 0.000* | 7.11 (2.1) | 0.000* |
| **20°m-20°b** | 6.05 (1.8) | 0.000* | 6.13 (1.9) | 0.000* | 6.93 (2.2) | 0.000* |
| **ANOVA for Repeated measures** | *F*(6.57, 348.43)=20.66, *p*=0.0 | | *F*(6.14, 325.51)=15.89, *p*=0.0 | | *F*(6.25, 331.38)=9.54, *p*=0.0 | |

Additional table S3: Descriptive data and statistical outcome for the effect of rotation on lesion area [mm] for the incisors (n=54).
