# Supplementary material for: The reproducibility of assessment of white spot lesions adjacent to orthodontic brackets, with a quantitative light induced fluorescence digital camera at different rotations of teeth – an in vitro study
Source: BMC Oral Health. 2018 Dec 11;18:209. doi: 10.1186/s12903-018-0667-3 (PMC6290521; doi:10.1186/s12903-018-0667-3)
Supplement: Supplementary file 4 — Table S4. Descriptive data and statistical outcome for the effect of rotation on lesion area [mm] for the canines (WB: n=31, WE: n=30, AD: n=31 ). (DOCX 15 kb) [file 12903_2018_667_MOESM4_ESM.docx]

| **Rotation Angle** | **WB area** | | **WE area** | | **AD area** | |
| --- | --- | --- | --- | --- | --- | --- |
|  | **Mean (sd)** | **p-value comparison with 0°** | **Mean (sd)** | **p-value comparison with 0°** | **Mean (sd)** | **p-value comparison with 0°** |
| **20°d-20°l** | 4.64 (1.0) | 0.161 | 4.55 (0.9) | 0.005* | 5.49 (1.2) | 0.000* |
| **20°d-10°l** | 4.38 (1.1) | 0.000* | 4.54 (1.0) | 0.001* | 5.51 (1.2) | 0.000* |
| **20°d-0°bl** | 4.35 (1.1) | 0.000* | 4.40 (1.1) | 0.000* | 5.81 (1.2) | 0.003* |
| **20°d-10°b** | 4.27 (1.0) | 0.000* | 4.16 (1.1) | 0.000* | 5.82 (1.3) | 0.003* |
| **20°d-20°b** | 3.95 (1.0) | 0.000* | 3.77 (1.0) | 0.000* | 5.73 (1.3) | 0.001* |
| **10°d-20°l** | 4.83 (1.0) | 0.887 | 4.83 (1.1) | 0.806 | 5.76 (1.1) | 0.003 |
| **10°d-10°l** | 4.74 (1.2) | 0.240 | 4.67 (1.2) | 0.039* | 5.89 (1.3) | 0.008* |
| **10°d-0°bl** | 4.81 (1.1) | 0.753 | 4.62 (1.3) | 0.007* | 6.10 (1.4) | 0.141 |
| **10°d-10°b** | 4.46 (1.2) | 0.001* | 4.47 (1.2) | 0.000* | 6.11 (1.4) | 0.080 |
| **10°d-20°b** | 4.16 (1.0) | 0.000* | 4.00 (1.2) | 0.000* | 6.12 (1.3) | 0.200 |
| **0°md-20°l** | 4.95 (1.1) | 0.353 | 4.94 (1.1) | 0.267 | 5.94 (1.3) | 0.001* |
| **0°md-10°l** | 4.87 (1.2) | 0.735 | 4.99 (1.2) | 0.020* | 6.11 (1.4) | 0.014* |
| **0°** | 4.84 (1.4) | - | 4.85 (1.2) | - | 6.29 (1.5) | - |
| **0°md-10°b** | 4.65 (1.3) | 0.032* | 4.59 (1.3) | 0.000* | 6.29 (1.5) | 0.934 |
| **0°md-20°b** | 4.29 (1.1) | 0.000* | 4.25 (1.2) | 0.000* | 6.28 (1.4) | 0.952 |
| **10°m-20°l** | 4.70 (1.1) | 0.162 | 4.74 (1.2) | 0.318 | 5.79 (1.3) | 0.000* |
| **10°m-10°l** | 4.73 (1.2) | 0.126 | 4.67 (1.2) | 0.074 | 5.80 (1.4) | 0.000* |
| **10°m-0°bl** | 4.69 (1.2) | 0.047* | 4.69 (1.2) | 0.017* | 6.09 (1.4) | 0.038* |
| **10°m-10°b** | 4.50 (1.1) | 0.001* | 4.55 (1.3) | 0.001* | 6.10 (1.4) | 0.061 |
| **10°m-20°b** | 4.28 (1.1) | 0.000* | 4.16 (1.1) | 0.000 | 5.95 (1.4) | 0.016* |
| **20°m-20°l** | 4.61 (1.1) | 0.080 | 4.62 (1.1) | 0.090 | 5.35 (1.1) | 0.000* |
| **20°m-10°l** | 4.61 (1.2) | 0.002* | 4.54 (1.2) | 0.004* | 5.46 (1.4) | 0.000* |
| **20°m-0°bl** | 4.78 (1.1) | 0.368 | 4.65 (1.1) | 0.072 | 5.74 (1.4) | 0.000* |
| **20°m-10°b** | 4.57 (1.0) | 0.019* | 4.54 (1.1) | 0.016* | 5.73 (1.3) | 0.000* |
| **20°m-20°b** | 4.34 (1.0) | 0.000* | 4.14 (1.1) | 0.000* | 5.60 (1.3) | 0.000* |
| **ANOVA for Repeated measures** | *F*(6.77, 202.95)=12.01, *p*=0.0 | | *F*(5.77, 167.17)=16.23, *p*=0.0 | | *F*(5.97, 178.98)=11.07, *p*=0.0 | |

Additional table S4: Descriptive data and statistical outcome for the effect of rotation on lesion area [mm] for the canines (WB: n=31, WE: n=30, AD: n=31 ).
